# Cytostatic and Non-Apoptotic Effects of Vinorelbine-Based Therapy in 3D Endometrial Cancer Spheroids

**DOI:** 10.3390/biology15070576

**Published:** 2026-04-03

**Authors:** Berna Yıldırım, Burcu Biltekin, Mete Hakan Karalök, Ayhan Bilir

**Affiliations:** 1 Department of Histology and Embryology, Faculty of Medicine, İstanbul Atlas University, 34403 İstanbul, Turkey; berna.yildirim@atlas.edu.tr (B.Y.); burcu.biltekin@atlas.edu.tr (B.B.); 2 Department of Obstetrics and Gynecology, Faculty of Medicine, İstanbul Atlas University, 34403 İstanbul, Turkey; hakan.karalok@atlas.edu.tr

**Keywords:** endometrial cancer, vinorelbine, three-dimensional spheroids, cytostasis, cell cycle arrest, non-apoptotic mechanisms, lithium chloride, medroxyprogesterone acetate

## Abstract

Resistance to apoptosis represents a major therapeutic challenge in advanced and recurrent endometrial cancer. This study investigated whether vinorelbine, alone or in combination with lithium chloride (LiCl) or medroxyprogesterone acetate (MPA), suppresses tumor growth through non-apoptotic mechanisms in a three-dimensional spheroid model of Ishikawa endometrial cancer cells. Proliferation was assessed by BrdU incorporation, cell cycle distribution by flow cytometry, and apoptosis by Annexin V/propidium iodide staining. Vinorelbine-based treatments significantly reduced DNA synthesis and were associated with decreased S-phase fractions and altered cell cycle distribution. These effects were sustained over time and were more pronounced in combination treatments. Importantly, no consistent increase in apoptotic cell death was observed, and most cells remained viable despite strong suppression of proliferation. These findings indicate that vinorelbine-based regimens inhibit tumor spheroid growth predominantly through cytostatic mechanisms rather than induction of apoptosis. This non-apoptotic growth-control strategy may be relevant for the management of apoptosis-resistant endometrial cancer.

## Simple Summary

Endometrial cancer is one of the most common gynecological cancers, and treatment can be challenging when tumor cells become resistant to therapies that rely on apoptosis (programmed cell death). In this study, we used a three-dimensional tumor model to better mimic real tumor behavior and investigated the effects of vinorelbine, alone or combined with lithium chloride or medroxyprogesterone acetate. We found that these treatments significantly reduced tumor cell proliferation without strongly inducing cell death. Instead, the drugs appeared to slow down or stop tumor growth by suppressing DNA synthesis and altering cell cycle progression. These findings suggest that some anticancer therapies may control tumor growth without necessarily killing the cells, which could be particularly useful in apoptosis-resistant cancers. This approach may help guide the development of alternative treatment strategies for patients with advanced or recurrent endometrial cancer.

## 1. Introduction

Endometrial carcinoma is the most common gynecologic malignancy in developed countries, and its incidence has increased steadily over recent decades [1,2]. Although early-stage disease is often curable by surgery with or without adjuvant therapy, advanced or recurrent endometrial cancer remains difficult to treat and is associated with poor prognosis [3,4]. Standard first-line chemotherapy, typically carboplatin–paclitaxel, achieves objective responses in approximately 50–60% of patients; however, durable remissions are uncommon and treatment-related toxicity is substantial [5]. Hormonal therapy with progestins represents a well-tolerated option for a subset of hormone receptor–positive tumors, yet response rates remain limited in unselected patient populations [5].

A major biological barrier to durable therapeutic success in advanced endometrial cancer is resistance, frequently driven by the tumor’s ability to evade apoptosis. This resistance is often associated with TP53 alterations and upregulation of anti-apoptotic signaling pathways, significantly limiting the efficacy of both cytotoxic and targeted agents [6]. Consequently, increasing attention has focused on alternative therapeutic strategies that suppress tumor growth through non-apoptotic mechanisms, such as sustained cytostatic responses, autophagy-associated stress pathways, or other non-apoptotic growth-control states [6,7]. As a result, there is increasing interest in alternative, non-apoptotic cancer therapies that can arrest tumor growth or induce cell death through regulated mechanisms such as autophagy, senescence, or necroptosis [8]. These approaches bypass caspase-dependent apoptosis and may offer advantages in apoptosis-resistant tumors, while potentially engaging immune-mediated clearance mechanisms [8]. Among non-apoptotic outcomes, therapy-induced cytostatic states, characterized by durable cell cycle arrest in metabolically active cells, have gained considerable interest [9]. In contrast, classical apoptotic cell death is often immunologically silent [10,11,12]. Thus, induction of non-apoptotic growth arrest has emerged as a promising strategy to circumvent apoptosis resistance and achieve sustained tumor control.

Within this context, vinorelbine, a semi-synthetic vinca alkaloid that disrupts microtubule dynamics and is clinically used in breast and non-small cell lung cancers, has shown context-dependent biological effects [13,14]. In addition to its cytotoxic activity at higher doses, vinorelbine has been reported to promote durable non-proliferative states, including non-proliferative cytostatic states, depending on dose and cellular context [15,16]. Preclinical studies have linked low-dose vinorelbine to upregulation of p21^Cip1/Waf1, retinoblastoma protein dephosphorylation, and suppression of E2F-driven cell cycle progression, resulting in cell cycle modulation and suppression of proliferative progression [16,17,18]. These observations suggest that vinorelbine may exert growth-suppressive effects even in settings where apoptotic pathways are attenuated.

To further enhance cytostatic efficacy, combination strategies targeting complementary growth-regulatory pathways are being explored. Lithium chloride (LiCl), a clinically used inhibitor of glycogen synthase kinase-3β, has demonstrated anti-proliferative activity in several cancer models, including endometrial cancer, by enforcing G_0_/G_1_ arrest and modulating autophagy-related stress responses without inducing apoptosis [19,20,21,22]. Similarly, medroxyprogesterone acetate (MPA), a synthetic progestin widely used in endometrial disease, suppresses proliferation through progesterone receptor–mediated regulation of cell cycle regulators, leading to robust G_0_/G_1_ arrest [23,24]. MPA has also been shown to enhance the growth-inhibitory effects of other agents in endometrial cancer models [25].

Based on this rationale, the present study aimed to systematically investigate whether vinorelbine, alone or in combination with LiCl or MPA, suppresses endometrial cancer cell proliferation through non-apoptotic mechanisms. Using a three-dimensional (3D) Ishikawa endometrial carcinoma spheroid model, we evaluated proliferation, cell cycle dynamics, and apoptotic status to determine whether these treatments enforce a sustained cytostatic, non-proliferative state. Our findings indicate that vinorelbine-based combination regimens effectively restrict tumor spheroid growth predominantly through sustained suppression of proliferative activity rather than induction of apoptosis, highlighting a non-apoptotic growth-control strategy with potential relevance for apoptosis-resistant endometrial cancer.

## 2. Materials and Methods

### 2.1. Cell Line and Culture Conditions

The human endometrial adenocarcinoma cell line Ishikawa (obtained from the European Collection of Authenticated Cell Cultures (ECACC) and purchased from Sigma-Aldrich (St. Louis, MO, USA)) was used as an in vitro model of well-differentiated, estrogen- and progesterone receptor–positive type I endometrial carcinoma. Cells were maintained in phenol red–free RPMI-1640 medium supplemented with 10% fetal bovine serum (FBS), 2 mM L-glutamine, 100 U/mL penicillin, and 100 µg/mL streptomycin. Cultures were incubated at 37 °C in a humidified atmosphere containing 5% CO_2_. Low-passage cells (≤15 passages) were used throughout the study to ensure phenotypic stability.

### 2.2. Three-Dimensional (3D) Spheroid Culture

For three-dimensional culture, Ishikawa cells were seeded into ultra-low attachment (ULA) 96-well plates (Corning, Corning, NY, USA) to enable spontaneous spheroid formation. Single-cell suspensions were prepared from semi-confluent monolayers, and 5 × 10^3^ viable cells were plated per well in complete medium. Plates were incubated under standard culture conditions, allowing for the formation of compact multicellular spheroids within 48 h. Spheroid morphology and structural integrity were confirmed by phase-contrast microscopy prior to initiation of drug treatments. Wells were assigned to treatment groups in a systematic and balanced manner to minimize positional effects within plates.

### 2.3. Drug Treatment

Vinorelbine (VNR), lithium chloride (LiCl), and medroxyprogesterone acetate (MPA) were used for experimental treatments. Stock solutions were prepared according to the manufacturers’ recommendations, with LiCl dissolved in sterile distilled water and MPA in dimethyl sulfoxide (DMSO). Working solutions were freshly prepared in complete culture medium immediately before use. Cells were treated with vinorelbine (10 nM), lithium chloride (10 mM), or medroxyprogesterone acetate (200 µM). These concentrations were selected based on previous reports and preliminary titration experiments demonstrating cytostatic effects without overt cytotoxicity, consistent with prior studies investigating LiCl- and MPA-mediated growth suppression in endometrial and other cancer models [19,23,24]. The selected concentrations were intended to induce measurable biological responses in a 3D spheroid context rather than to directly reflect plasma-equivalent clinical dosing. Control groups included both untreated spheroids and vehicle controls corresponding to each treatment condition (distilled water for LiCl and DMSO for MPA), with the final DMSO concentration not exceeding 0.1% (*v*/*v*).

Drug treatments were initiated 48 h after spheroid seeding, once uniform spheroids had formed, and this time point was defined as 0 h. Experimental groups included untreated control spheroids, vinorelbine monotherapy, and combination treatments consisting of vinorelbine plus LiCl or vinorelbine plus MPA. Single-agent LiCl and MPA groups were not included, as the primary aim was to evaluate whether these agents enhance vinorelbine-induced cytostatic effects. Triple combination treatments were also not included to allow clear interpretation of the contribution of each adjunct agent. Treatments were applied for 24, 48, 72, and 96 h. All experiments were independently repeated at least three times to ensure reproducibility.

### 2.4. BrdU Proliferation Assay

Cell proliferation within 3D spheroids was assessed using a colorimetric 5-bromo-2′-deoxyuridine (BrdU) incorporation assay (Roche Diagnostics, Basel, Switzerland). At defined time points (24, 48, 72, and 96 h after treatment), BrdU labeling solution was added to the culture medium at a final concentration of 10 µM for the last 2 h of incubation. Following labeling, spheroids were harvested and enzymatically dissociated into single-cell suspensions using trypsin–EDTA with gentle mechanical trituration. Cells were fixed, and incorporated BrdU was detected using a peroxidase-conjugated anti-BrdU antibody according to the manufacturer’s protocol. Absorbance was measured at 370 nm with a reference wavelength of 492 nm using a microplate reader. BrdU incorporation was normalized to untreated control spheroids. Each condition was analyzed in six technical replicates per experiment, and all experiments were performed in biological triplicates.

### 2.5. Cell Cycle Analysis

Cell cycle distribution was evaluated by flow cytometry following propidium iodide (PI) staining. At 24, 48, 72, and 96 h after treatment, spheroids were collected and dissociated into single-cell suspensions. Cells were washed with phosphate-buffered saline (PBS) and fixed in 70% cold ethanol for a minimum of 2 h, at −20 °C. After fixation, cells were washed, treated with RNase A (50 µg/mL, 30 min at 37 °C), and stained with PI (50 µg/mL) in PBS containing 0.1% Triton X-100. DNA content was analyzed using a BD FACSCanto II flow cytometer (BD Biosciences, San Jose, CA, USA), acquiring at least 20,000 events per sample. Doublets were excluded by pulse-geometry gating. Cell cycle phase distribution (G_0_/G_1_, S, and G_2_/M) was quantified using FlowJo v10 software. All analyses were performed in triplicate. Data acquisition and analysis were performed using standardized gating strategies and analysis settings applied uniformly across all samples to ensure consistency and minimize operator-dependent variability.

### 2.6. Annexin V-FITC/PI Apoptosis Assay

Apoptotic and necrotic cell populations were assessed using dual Annexin V-FITC and propidium iodide (PI) staining followed by flow cytometry. At 24, 48, 72, and 96 h after treatment, spheroids were dissociated into single-cell suspensions, washed with PBS, and resuspended in binding buffer at a concentration of approximately 1 × 10^6^ cells/mL. Annexin V-FITC (5 µL) and PI (5 µL) were added to 100 µL of cell suspension and incubated for 15 min at room temperature in the dark. After the addition of binding buffer, samples were immediately analyzed using a BD FACSCanto II flow cytometer (BD Biosciences, San Jose, CA, USA). At least 10,000 events were acquired per sample. Cells were classified as viable, early apoptotic, or late apoptotic/necrotic based on Annexin V and PI staining patterns. Data were analyzed using FlowJo software. All experiments were independently repeated three times.

### 2.7. Statistical Analysis

All quantitative data are presented as mean ± standard deviation (SD) from three independent biological replicates. For proliferation assays, each condition included six technical replicates per experiment. Prior to statistical analysis, data distribution was assessed for normality using the Shapiro–Wilk test. For normally distributed data, comparisons were performed using parametric tests as described below. BrdU proliferation data were analyzed using two-way analysis of variance (ANOVA) followed by Dunnett’s post hoc test for multiple comparisons against the control group. Cell cycle and apoptosis data were analyzed using one-way ANOVA followed by Tukey’s post hoc test at each time point. Exact *p*-values are reported where applicable, and a *p*-value < 0.05 was considered statistically significant. All statistical analyses were performed using GraphPad Prism version 9 (GraphPad Software, San Diego, CA, USA). Effect sizes were consistent across independent experiments, supporting the robustness of the observed trends.

## 3. Results

**Figure 1:**
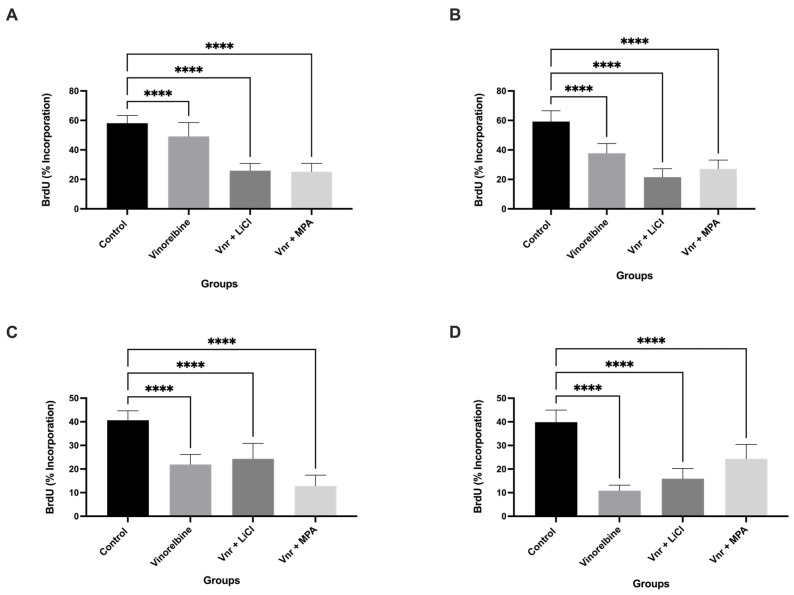
**Vinorelbine-based treatments inhibit proliferation of 3D Ishikawa spheroids.** BrdU incorporation (% positive cells) was measured at 24 h (**A**), 48 h (**B**), 72 h (**C**), and 96 h (**D**) following treatment with vinorelbine, vinorelbine + LiCl, or vinorelbine + MPA. Data represent mean ± SD of three independent experiments. Statistical significance: **** *p* < 0.0001 vs. control.

Cell proliferation in Ishikawa 3D spheroids was evaluated by bromodeoxyuridine (BrdU) incorporation at 24, 48, 72, and 96 h following treatment with vinorelbine (VNR) alone or in combination with lithium chloride (LiCl) or medroxyprogesterone acetate (MPA) (Figure 1A–D). At 24 h (Figure 1A), VNR treatment resulted in a significant reduction in BrdU incorporation compared with untreated controls, while both combination treatments were associated with a more pronounced decrease compared to vinorelbine alone. This suppressive effect on DNA synthesis persisted over time. At 48 and 72 h (Figure 1B,C), BrdU incorporation was markedly reduced across all treatment groups, with combination treatments generally showing stronger suppression compared to vinorelbine alone. However, the extent of reduction varied between LiCl and MPA combinations depending on the time point. By 96 h (Figure 1D), BrdU positivity declined further across all groups, reaching approximately 10–25% in treated spheroids. Notably, vinorelbine alone also exhibited a strong suppressive effect at later time points. These findings indicate that vinorelbine, both alone and in combination with LiCl or MPA, induces sustained suppression of proliferative activity in 3D endometrial cancer spheroids.

**Figure 2:**
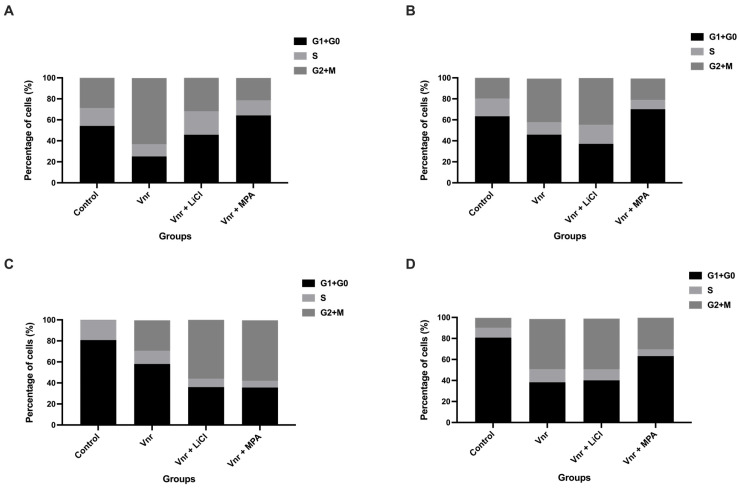
**Vinorelbine-based treatments induce cell cycle redistribution in 3D Ishikawa spheroids.** (**A**–**D**) Representative flow cytometry plots showing the distribution of cells in G_0_/G_1_, S, and G_2_/M phases at 24 h (**A**), 48 h (**B**), 72 h (**C**), and 96 h (**D**) post-treatment with vinorelbine, vinorelbine + LiCl, or vinorelbine + MPA. Data shown are from one of three independent experiments.

To determine whether the observed growth suppression was accompanied by alterations in cell cycle progression, DNA content analysis was performed on spheroid-derived cells at 24, 48, 72, and 96 h following treatment (Figure 2A–D). At 24 h (Figure 2A), only the VNR + MPA group exhibited a modest increase in the G_0_/G_1_ fraction, whereas VNR alone and VNR + LiCl showed a relative reduction compared with the control. This pattern evolved over time in a non-uniform manner. At 48 h (Figure 2B), the VNR + MPA group exhibited the highest G_0_/G_1_ fraction, whereas VNR alone and VNR + LiCl showed reduced levels compared to the control. At later time points (72 and 96 h; Figure 2C,D), G_0_/G_1_ fractions in all treatment groups were generally lower than in control spheroids, indicating a redistribution of cells across the cell cycle rather than uniform G_0_/G_1_ accumulation. In parallel, S-phase fractions consistently declined across all treatment conditions, particularly in combination groups, supporting suppression of DNA synthesis. The G_2_/M fraction showed variable but non-dominant changes across groups. Overall, these findings suggest that vinorelbine-based treatments induce complex alterations in cell cycle dynamics, characterized primarily by suppression of S-phase entry rather than sustained G_0_/G_1_ accumulation.

**Figure 3:**
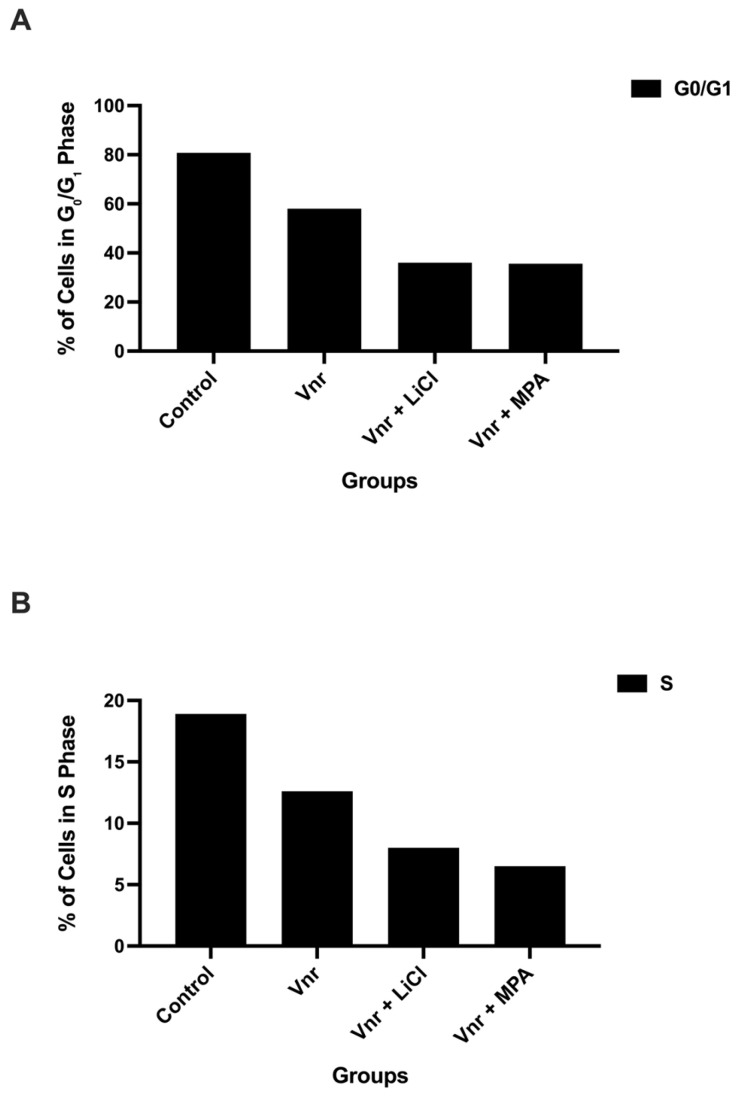
**Quantitative analysis of cell cycle alterations induced by vinorelbine-based treatments.** (**A**) Percentage of spheroid-derived cells in G_0_/G_1_ phase at 72 h. (**B**) Percentage of cells in S phase at 72 h. Vinorelbine-based treatments are associated with reduced S-phase fractions and altered cell cycle distribution. Data represent mean ± SD from three independent experiments.

To further substantiate the cytostatic effects observed, quantitative analysis of cell cycle distribution was performed at 72 h (Figure 3A,B). The proportion of cells in the G_0_/G_1_ phase was highest in control spheroids and decreased across all treatment groups, with the lowest levels observed in the VNR + LiCl and VNR + MPA combinations. In contrast, S-phase populations were markedly reduced in all treated groups, declining to approximately 5–10%, particularly in combination treatments. Vinorelbine monotherapy produced intermediate effects, with partial reductions in S-phase entry. These quantitative findings indicate that vinorelbine-based treatments are associated with suppression of DNA synthesis and redistribution of cell cycle phases rather than a uniform increase in G_0_/G_1_ accumulation.

**Figure 4:**
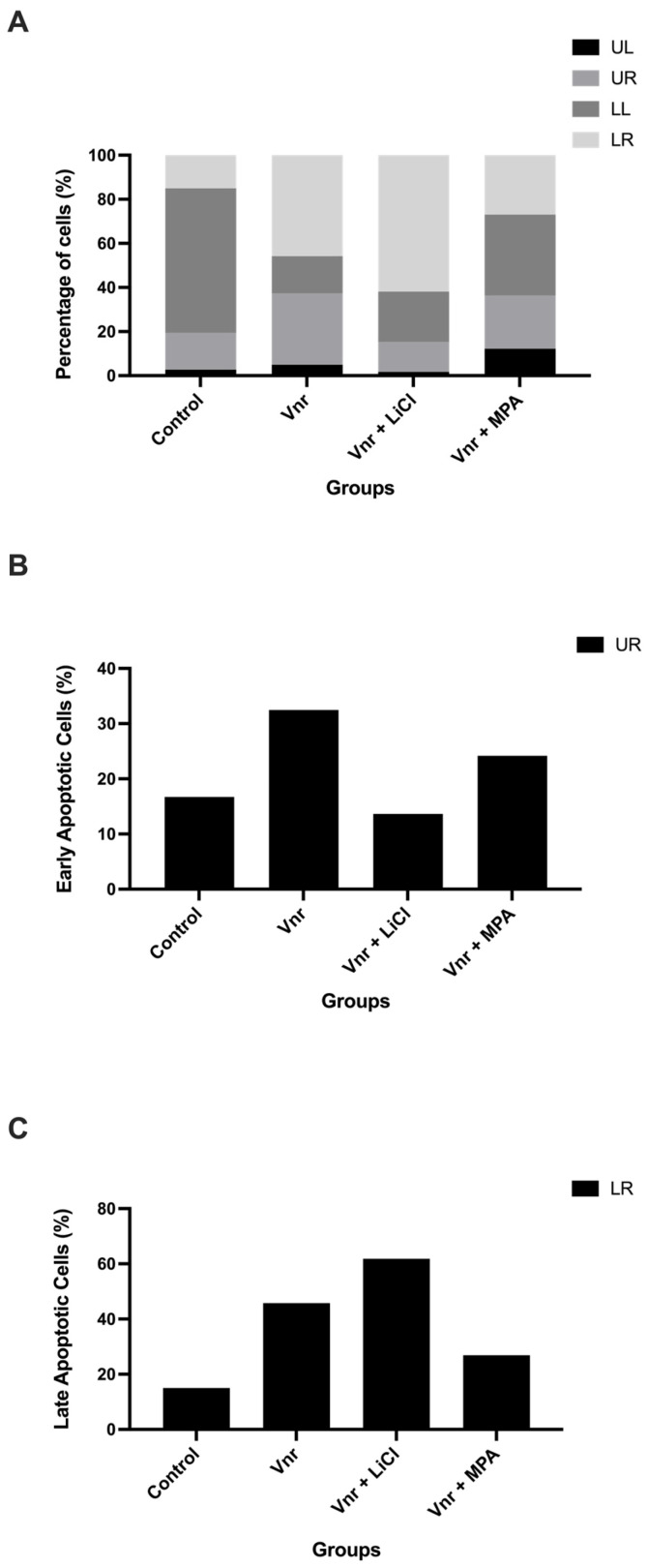
**Vinorelbine-based treatments do not prominently induce apoptosis in 3D Ishikawa spheroids at 72 h.** (**A**) Flow cytometry-based distribution of viable (LL), early apoptotic (LR), and late apoptotic/necrotic (UR) cells following treatment. (**B**) Quantification of late apoptotic (UR) cells. (**C**) Quantification of early apoptotic (LR) cells. Data represent mean ± SD from three independent experiments.

To assess whether growth suppression was associated with apoptotic or necrotic cell death, Annexin V–FITC/propidium iodide (PI) staining was performed on spheroid-derived cells at 72 h following treatment (Figure 4A–C). Across all experimental groups, the majority of cells remained viable (Annexin V^−^/PI^−^), with variable but non-dominant changes observed in early apoptotic (Annexin V^+^/PI^−^; LR) and late apoptotic/necrotic (Annexin V^+^/PI^+^; UR) populations compared with untreated controls. Quantitative analysis showed that vinorelbine treatment, either alone or in combination with LiCl or MPA, did not result in a consistent or statistically significant increase in apoptotic cell fractions across groups. Although some variability in early and late apoptotic populations was observed, these changes were not indicative of a dominant apoptotic response. These findings indicate that vinorelbine-based growth suppression in 3D spheroids occurs predominantly in the absence of robust classical apoptotic cell death, supporting a primarily cytostatic mechanism. Representative flow cytometry plots are provided in Supplementary Figure S1.

## 4. Discussion

In this study, we examined the effects of vinorelbine, administered alone or in combination with lithium chloride (LiCl) or medroxyprogesterone acetate (MPA), on proliferation and cell fate in 3D endometrial cancer spheroids. Our findings demonstrate that all treatment conditions were associated with a substantial reduction in BrdU incorporation, reflecting a marked suppression of DNA synthesis. This antiproliferative response was accompanied by a consistent reduction in S-phase populations and alterations in cell cycle distribution rather than uniform accumulation in a single phase. In parallel, Annexin V analysis revealed no consistent increase in apoptotic cell death across treatment groups. Together, these observations indicate that vinorelbine-based regimens primarily limit spheroid growth through non-apoptotic, cytostatic mechanisms rather than activation of classical apoptotic pathways. Within the context of a 3D tumor model, these findings support the concept that sustained suppression of proliferative activity may represent an effective strategy in settings where apoptotic responsiveness is limited [6,8]. It should be noted that the concentrations used in this study are higher than typical physiological levels, which is a recognized limitation of in vitro models. These doses were selected to achieve measurable biological responses in a 3D spheroid system rather than to directly reflect clinical exposure levels. Therefore, further studies will be required to define clinically relevant dosing windows and to evaluate translational applicability

These findings are consistent with previous reports demonstrating that vinorelbine, a microtubule-targeting agent, can exert context-dependent biological effects, including induction of non-proliferative states in addition to apoptosis [13,15]. While therapy-induced senescence has been described in association with microtubule-targeting agents [18], the present study does not directly assess senescence-specific markers. Therefore, the observed phenotype is more appropriately interpreted as a cytostatic, non-proliferative state characterized by suppression of DNA synthesis. Importantly, the enhanced S-phase depletion observed in combination treatments suggests that LiCl and MPA may potentiate vinorelbine-induced cytostatic effects. Previous studies have shown that LiCl and MPA can modulate cell cycle regulators and growth-related signaling pathways in endometrial cancer models [19,23,24,25]. Taken together, these findings support the concept that targeting complementary regulatory pathways may enhance cytostatic responses in apoptosis-resistant tumor contexts.

In breast cancer models, low-dose vinorelbine has been associated with non-proliferative cellular states and regulation of cell cycle progression [14,25]. While previous studies have linked vinorelbine to senescence-associated pathways, the current study does not directly assess senescence markers such as SA-β-gal activity or SASP factors. Therefore, the observed phenotype is more appropriately interpreted as a cytostatic, non-proliferative state rather than definitive therapy-induced senescence.

LiCl, a GSK-3β inhibitor, and MPA, a synthetic progestin, are both known to modulate cell cycle regulatory pathways through distinct mechanisms [26,27]. For example, LiCl-mediated GSK-3β inhibition has been reported to alter cell cycle progression in multiple cancer cell types [26]. Similarly, progestins such as MPA have been shown to suppress proliferation by reducing S-phase entry and modulating cell cycle regulators in hormone-responsive cancers [27,28]. Taken together, these findings suggest that combining vinorelbine with LiCl or MPA may engage complementary regulatory pathways, resulting in enhanced cytostatic effects consistent with the suppression of proliferative activity observed in our model.

A particularly notable aspect of our study is the clear dissociation between proliferative arrest and apoptosis. Despite strong inhibition of proliferation and suppression of proliferative activity, none of the treatments resulted in a consistent increase in apoptotic cell death, as confirmed by Annexin V-FITC/PI analysis. This distinction is clinically important in endometrial carcinoma, where therapeutic resistance due to apoptosis evasion, often associated with TP53 alterations or prior cytotoxic exposure, remains a major barrier [28,29]. Our data suggest that vinorelbine-based cytostatic regimens may represent a viable strategy for tumor growth control even in apoptosis-refractory disease, underscoring the therapeutic relevance of non-apoptotic approaches. Although Annexin V/PI staining is a widely accepted method for detecting early and late apoptosis, additional assays such as caspase activation or PARP cleavage were not performed. Therefore, while our data support a predominantly non-apoptotic response, we cannot fully exclude low-level or delayed apoptotic signaling.

Importantly, sustained suppression of proliferative activity may have biological consequences beyond immediate growth inhibition. In other tumor models, such responses have been associated with non-proliferative cellular states that can influence tumor behavior and microenvironmental interactions [30]. However, these aspects were not directly assessed in the present study and therefore remain speculative. Accordingly, we conservatively interpret the observed phenotype as durable cytostasis rather than definitive therapy-induced senescence. Future studies employing more complex or immune-competent models will be required to determine whether vinorelbine-induced cytostasis can engage additional tumor-control mechanisms in vivo.

We acknowledge several limitations of this study. First, our analyses were primarily focused on functional outcomes, including BrdU incorporation, cell cycle distribution, and apoptosis, and did not include molecular validation of cytostatic or senescence-associated pathways (such as SA-β-galactosidase activity, p16^INK4a, LC3, or p62). Therefore, while our findings support a predominantly cytostatic, non-apoptotic response, the precise molecular mechanisms underlying this phenotype remain to be elucidated. Second, the use of a single 3D spheroid model (Ishikawa, type I endometrial carcinoma) limits the generalizability of the findings, as endometrial cancer is a heterogeneous disease comprising both hormone-dependent and more aggressive, p53-mutant subtypes [31]. Validation in additional cell lines and in vivo systems will be required to confirm the broader applicability of these observations.

Importantly, the present study was designed as a functional and phenotypic characterization of vinorelbine-induced growth suppression in a 3D context rather than a detailed mechanistic dissection. Future studies incorporating molecular analyses will be essential to define the signaling pathways governing vinorelbine-induced cytostasis and to determine how these responses interact with tumor microenvironmental factors in more complex or immune-competent systems [32,33]. Taken together, our findings support further investigation of vinorelbine-based, non-apoptotic combination strategies as potential approaches for the management of apoptosis-resistant endometrial cancers.

## 5. Conclusions

In summary, our findings demonstrate that vinorelbine-based therapy, particularly in combination with lithium chloride (LiCl) or medroxyprogesterone acetate (MPA), suppresses the growth of endometrial cancer spheroids by reducing proliferative activity and altering cell cycle dynamics without activating classical apoptotic pathways. This predominantly cytostatic response supports the concept that non-apoptotic growth-control strategies may offer a complementary approach for tumors exhibiting resistance to apoptosis-dependent therapies. The effects observed in a 3D spheroid model highlight the relevance of non-apoptotic mechanisms in the context of advanced or recurrent endometrial cancer. Further studies employing in vivo and immune-competent models will be required to validate these findings and to define their potential translational relevance.

## Data Availability

The data supporting the findings of this study are included within the article and its Supplementary Materials. Additional datasets are available from the corresponding author upon reasonable request.

## Supplementary Materials

The following supporting information can be downloaded at: https://www.mdpi.com/article/10.3390/biology15070576/s1, Figure S1: Representative flow cytometry dot plots (Annexin V–FITC/PI) showing viable (LL), early apoptotic (LR), and late apoptotic/necrotic (UR) cell populations in Ishikawa 3D spheroids following treatment with vinorelbine (VNR), vinorelbine + lithium chloride (LiCl), or vinorelbine + medroxyprogesterone acetate (MPA) at 72 h.

## Abbreviations

The following abbreviations are used in this manuscript:

VNR	Vinorelbine
LiCl	Lithium Chloride
MPA	Medroxyprogesterone acetate
