# Supplementary figures and images for: Cytostatic and Non-Apoptotic Effects of Vinorelbine-Based Therapy in 3D Endometrial Cancer Spheroids
Source: Biology (Basel). 2026 Apr 3;15(7):576. doi: 10.3390/biology15070576 (PMC13071962; doi:10.3390/biology15070576)

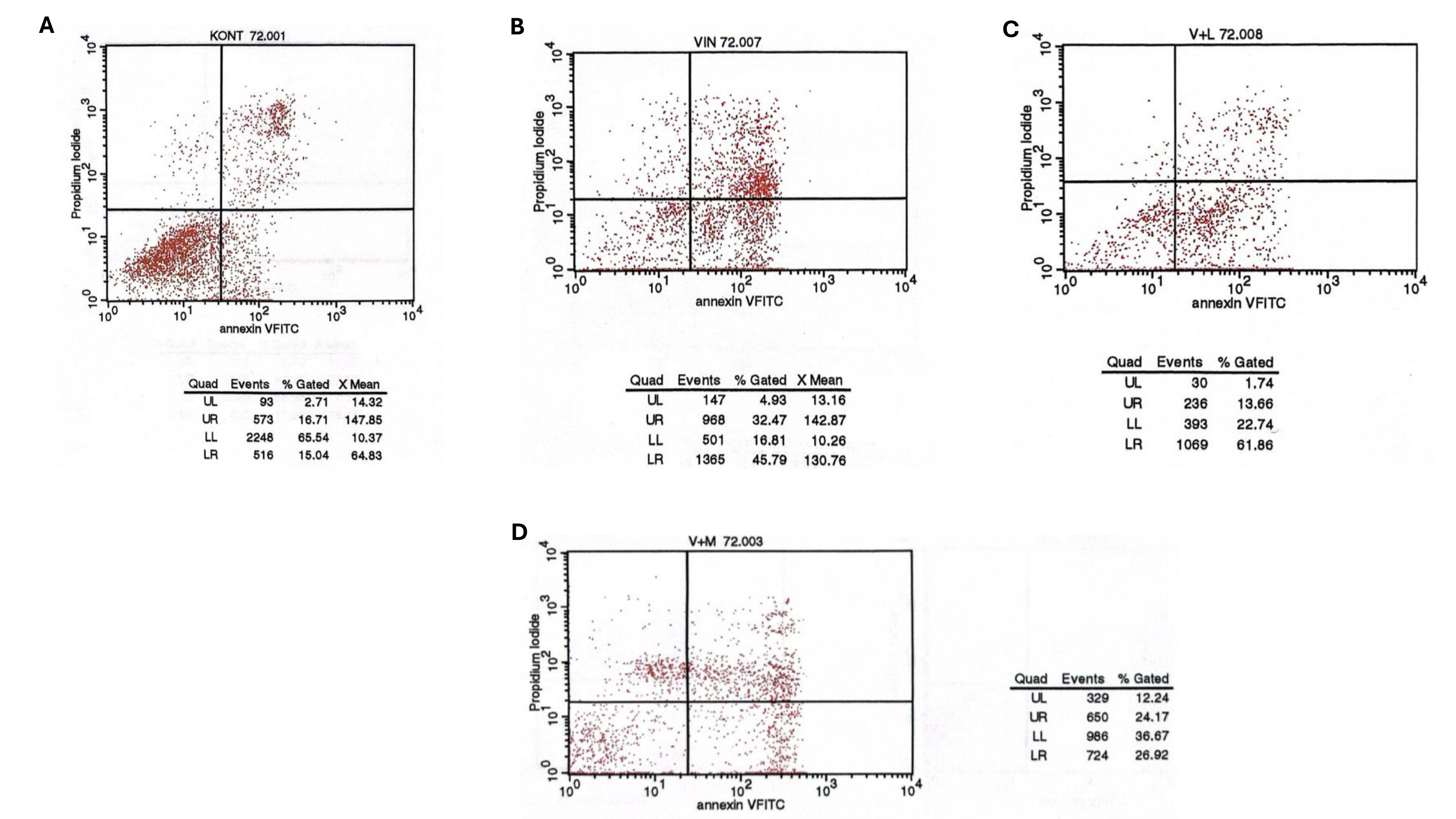

Supplement: Supplementary file 1 [file biology-15-00576-s001.zip › biology-4221193-supplementary.tiff]
